# Safety and efficacy of the novel motorized power spiral enteroscopy: A single‐center experience

**DOI:** 10.1002/deo2.148

**Published:** 2022-07-11

**Authors:** Pankaj Singh, Vikas Singla, Sawan Bopanna, Muzaffer Rashid Shawl, Pallavi Garg, Jatin Agrawal, Anupama Arya, Vibhuti Mittal, Richa Bhargava, Kaushal Madan

**Affiliations:** ^1^ Centre for Gastroenterology, Hepatology and Endoscopy Max Super Speciality Hospital New Delhi India; ^2^ Department of Pathology Max Super Speciality Hospital New Delhi India

**Keywords:** antegrade and retrograde enteroscopy, balloon‐assisted enteroscopy, pan‐enteroscopy, power spiral enteroscopy, small‐bowel diseases

## Abstract

**Objective:**

Evaluation and management of small bowel disorders have always been challenging due to the limitations of the existing technology. Motorized power spiral enteroscopy (PSE) is an innovative new technique that offers easier, faster, and more complete small bowel evaluation with the ability to perform therapeutics. We aimed to evaluate the safety and efficacy of PSE in various indications.

**Methods:**

Retrospective analysis of prospectively collected data of consecutive patients, who underwent PSE at a tertiary care center. Primary outcome measures were technical success rate, pan‐enteroscopy rate, diagnostic and therapeutic yield, and the secondary outcomes measures were the depth of maximal insertion, median insertion time, and adverse events.

**Results:**

Fifty‐four patients (mean age of 49.38 ± 16.26 years) underwent PSE for small bowel evaluation. Technical success rate was 95.55% (antegrade route) and 93.10% (retrograde route).  Pan‐enteroscopy rate is 46.29% and antegrade enteroscopy to the cecum was achieved in eight patients.  Overall diagnostic and therapeutic yields were 85.18% and 30.76%, respectively. The most common findings were ulcero‐stricturing lesions (51.92%) followed by vascular lesions (9.61%). The most common histopathologic diagnosis was Crohn's disease in 29.62%. Median depth of maximal insertion was 400 cm (range 150–550 cm; antegrade route) and 180 cm (range 50–350 cm; retrograde route). The median insertion time to depth of maximal insertion was 70 min (range 30–110 min; antegrade route) and 45 min (range 20–70 min; retrograde route).  PSE‐associated major adverse events occurred in one patient and minor adverse events were seen in 48.14%.

**Conclusion:**

PSE is a safe and effective modality for the evaluation of small bowel disorders with a high diagnostic yield.

## INTRODUCTION

The small intestine, because of its length and tortuosity, has always been difficult to access by the endoscopic method. The last couple of decades have seen significant advancement in the management of small bowel disorders.^1^ Wireless video capsule endoscopy was approved by the Food and Drug Administration in 2001 as a revolutionary new diagnostic tool for direct visualization of the entire small bowel.^2^ This technology, served its purpose as a useful non‐invasive diagnostic modality with good sensitivity, but the need for therapeutic interventions in the small bowel was still lacking. Deep enteroscopy systems using single and double balloon provides a good non‐surgical diagnostic and therapeutic option for small bowel lesions.^3^, ^4^ The spiral enteroscopy introduced in 2008, is a two‐operator technique requiring manual rotation of a spiral overtube to pleat the small bowel over the enteroscope.^5^ Long procedure time and lack of complete small bowel enteroscopy are the major limiting factors with these techniques. Novel motorized power spiral enteroscopy (PSE) is the latest innovative technology that works on the same principle of spiral enteroscopy, integrated with a user‐controlled motorized handle and a footswitch.^6^ This promising new technology has the potential to offer a quicker and complete evaluation of the small bowel. Limited data is available on the efficacy and safety of PSE in patients with small bowel disorders. Our study describes the preliminary single‐center experience with this new tool.

## METHODS AND MATERIALS

The present study is a retrospective analysis of data of all the patients who underwent PSE from February 2021 to January 2022 retrieved from the database. Demographic, clinical, radiological, previous endoscopic procedure details, and the findings on PSE were entered in the data sheet. Primary outcomes were technical success, total enteroscopy rate (TER), diagnostic and therapeutic yield, and the secondary outcomes were, depth of maximal insertion (DMI), median insertion time, and adverse events. Statistical analysis was done using SPSS version 1.0.0.1406 (IBM Corp., Armonk, NY, USA). Continuous variables were expressed as either mean (standard deviation) or median (range). Categorical variables were expressed as the number of patients and percentage.

**FIGURE 1:**
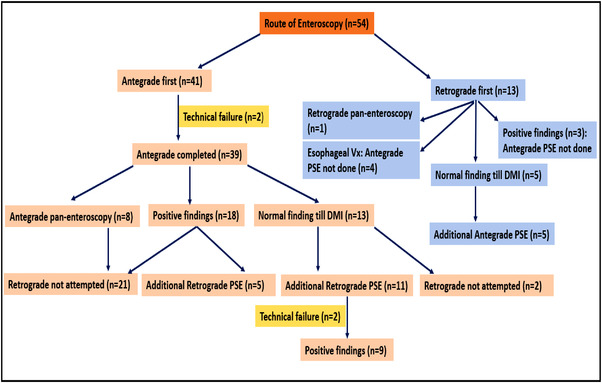
Flowchart showing an approach to small bowel evaluation using power spiral enteroscopy

Indications of the PSE were, unexplained chronic abdominal pain or small bowel obstruction with indeterminate findings on prior imaging, suspected small bowel Crohn's disease, suspected small bowel bleed, chronic diarrhea of unexplained etiology, therapeutic interventions in the mid‐small bowel, such as polypectomy, hemostasis, or stricture dilatation. The procedure was avoided in patients with severe coagulopathy or thrombocytopenia, recent abdominal surgeries/intestinal perforation, acute intestinal obstruction, multiple comorbidities not eligible for anesthesia, age less than 18 years, cervical disc prolapse, or inability to extend the neck, and pregnancy. Contraindications for the retrograde approach included severe inflammatory bowel disease in the colon, colonic stricture, and anal canal stenosis. In patients with esophageal stricture or suspected eosinophilic esophagitis, esophageal or gastric varices, decompensated advanced cirrhosis, retrograde evaluation was performed. The antegrade evaluation was preferred first, however in patients with imaging findings suggestive of the lesion within 150 cm of the ileocecal (IC) valve, the retrograde examination was performed first (Figure 1). Pan‐enteroscopy was not attempted in all patients. Retrograde enteroscopy was not done in patients with findings of non‐negotiable stricture or deep ulcers on antegrade enteroscopy which could likely explain the symptoms.

### Definitions

- Technical success rate (TSR): Successful passage of the enteroscope beyond the ligament of Treitz for antegrade procedures and retrograde approach successful negotiation of the enteroscope across the ileocecal valve.
- Pan‐enteroscopy rate (PER): Pan‐enteroscopy of the entire small bowel from the duodenojejunal flexure to the cecum, achieved either by the antegrade approach alone or combined antegrade and retrograde approaches.
- Partial enteroscopy: Non‐advancement of the enteroscope before reaching the target, failure to perform pan‐enteroscopy in case of non‐diagnostic findings, or in situations like tight strictures or deep ulcers the scope was intentionally not advanced further to avoid the risk of injury.
- DMI: Depth was estimated by the methods described previously.^7^, ^8^ Once the point where rotation of the PSE was ineffective in advancing the endoscope forward, was reached, a visual reference point at the tip of the scope (e.g., a circumferential fold) was identified. The same landmark was observed until an estimated withdrawal of 10 cm of the small bowel was performed, following which, a new reference point (another fold) was chosen. This process was repeated until the ligament of Treitz was reached for the antegrade approach and the ileocecal valve was reached for the retrograde approach, and the final distance was thus calculated.
- Diagnostic yield: Defined as the percentage of procedures with a positive yield for the findings that could explain the clinical symptoms
- Therapeutic success: Successful endoscopic interventions for bleeding, polypectomy, and stricture dilatation.
- Adverse events: Adverse events were categorized as minor or major. Superficial mucosal trauma like mild ooze, mucosal tear not amounting to perforation, esophageal pain and sore throat lasting less than 72 h and nausea, vomiting, or abdominal discomfort lasting less than 48 h were considered minor adverse events. Major adverse events were perforation, pancreatitis, aspiration pneumonitis, significant bleeding requiring blood transfusion, or any new or prolonged hospitalization related to the procedure complication.
- Successful procedure: Successful procedure was defined as either identification of the target area or pan enteroscopy in case of normal study.

### Procedure technique

**FIGURE 2:**
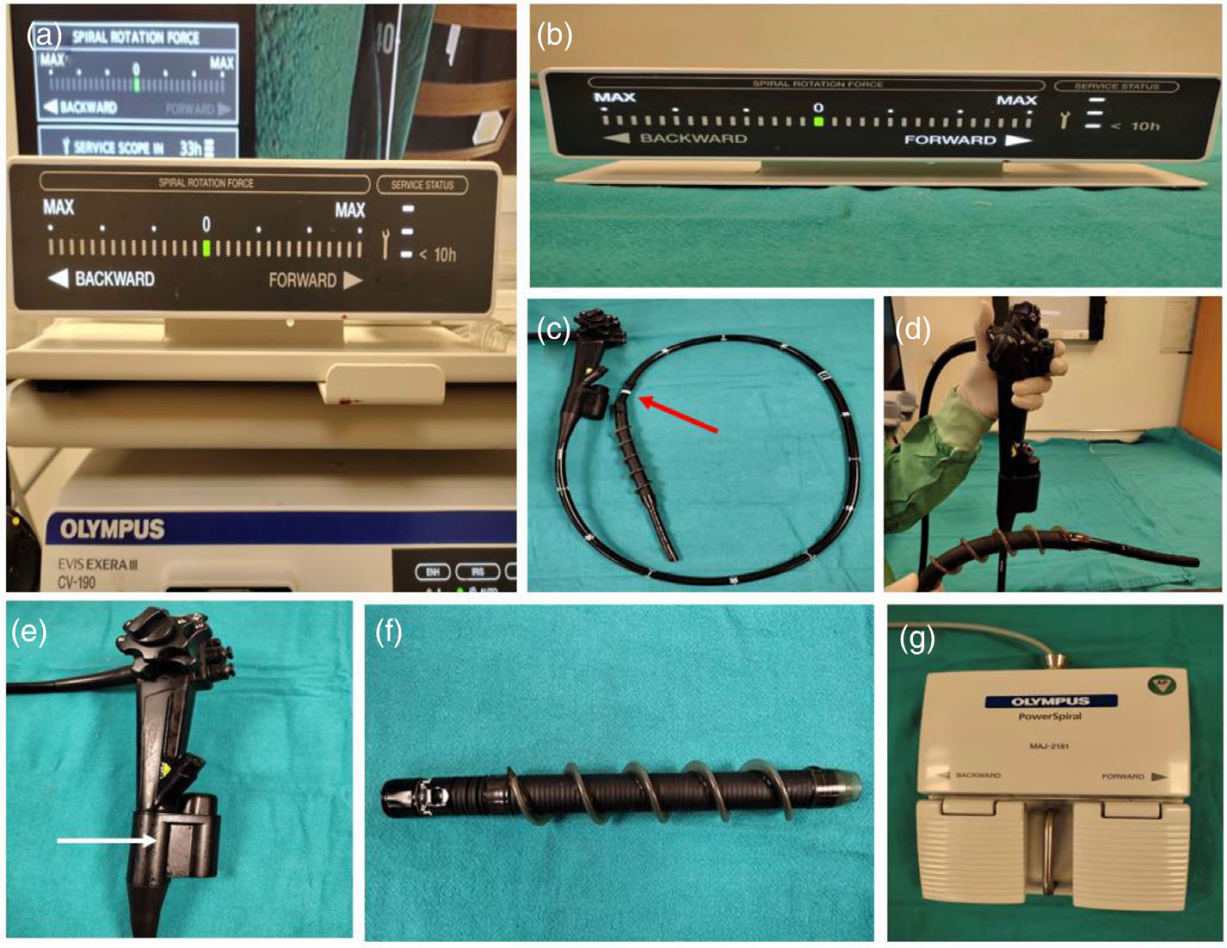
(a) Novel motorized power spiral enteroscopy system with power spiral (PSF‐1; Olympus Medical Systems, Tokyo, Japan). (b) A visual force gauge used to monitor the direction and force applied to the tissue by the spiral rotation of the enteroscope. (c–e) Reusable power spiral enteroscope with an integrated electric motor (white arrow); 1680 mm working length, 11.3 mm outer diameter (insertion portion), with 140° forward view, 180° up and down bending, and 160° left‐right movement, 3.2 mm accessory channel with dedicated separate irrigation channel. (f) Short disposable overtube with atraumatic soft spiral fins (240 mm length, 31.1 mm outer diameter of the spiral fins) attached to the rotation coupler (red arrow) located 40 cm proximal to the endoscope's tip. (g) Footswitch to control the direction and speed of motorized rotation of the spiral section

The technique of PSE has been previously described.^6^, ^9^ Novel motorized spiral enteroscopy has three basic components: (1) a reusable enteroscope with an integrated motor, (2) a short, disposable, power spiral overtube that is mounted on the insertion tube portion of the endoscope, and (3) a motorized power spiral control unit with a foot switch.^6^ Motorized control unit pleats and unpleats the small intestine over the spiral overtube (Figure 2). All antegrade procedures were done with the patient in the left lateral position under general anesthesia using nasotracheal intubation. Upper gastrointestinal endoscopy was performed in all the patients to rule out esophageal web or stricture. Serial bougie dilatation of the esophagus was done up to 20 mm to ensure easy passage of spiral tubes in patients with reduced esophageal compliance. The spiral rotation was started right at the cricopharyngeal region and continued as the spiral segment moves forward and pleats the bowel loops over the enteroscope. After reaching the ligament of treitz, carbon dioxide was switched off and the water immersion technique was used. The enteroscope was advanced until either the target lesion was reached or pan‐enteroscopy was performed. If the desired area could not be reached by the antegrade route, clip application was done to mark the site of maximal insertion and the retrograde approach was tried the same day. Pan‐enteroscopy was achieved using the antegrade approach alone or in a combination of antegrade and retrograde PSE/ileocolonoscopy/balloon‐assisted enteroscopy (BAE). The enteroscope needs to be withdrawn gradually by anticlockwise rotation; the enteroscope position has to be maintained at 80 cm until it exits from the pylorus. Sites and degree of mucosal trauma were noted on withdrawal, in cases with deep injury endoscopic therapy was done at the same time. Written informed consent was obtained from all patients before the procedure

## RESULTS

**FIGURE 3:**
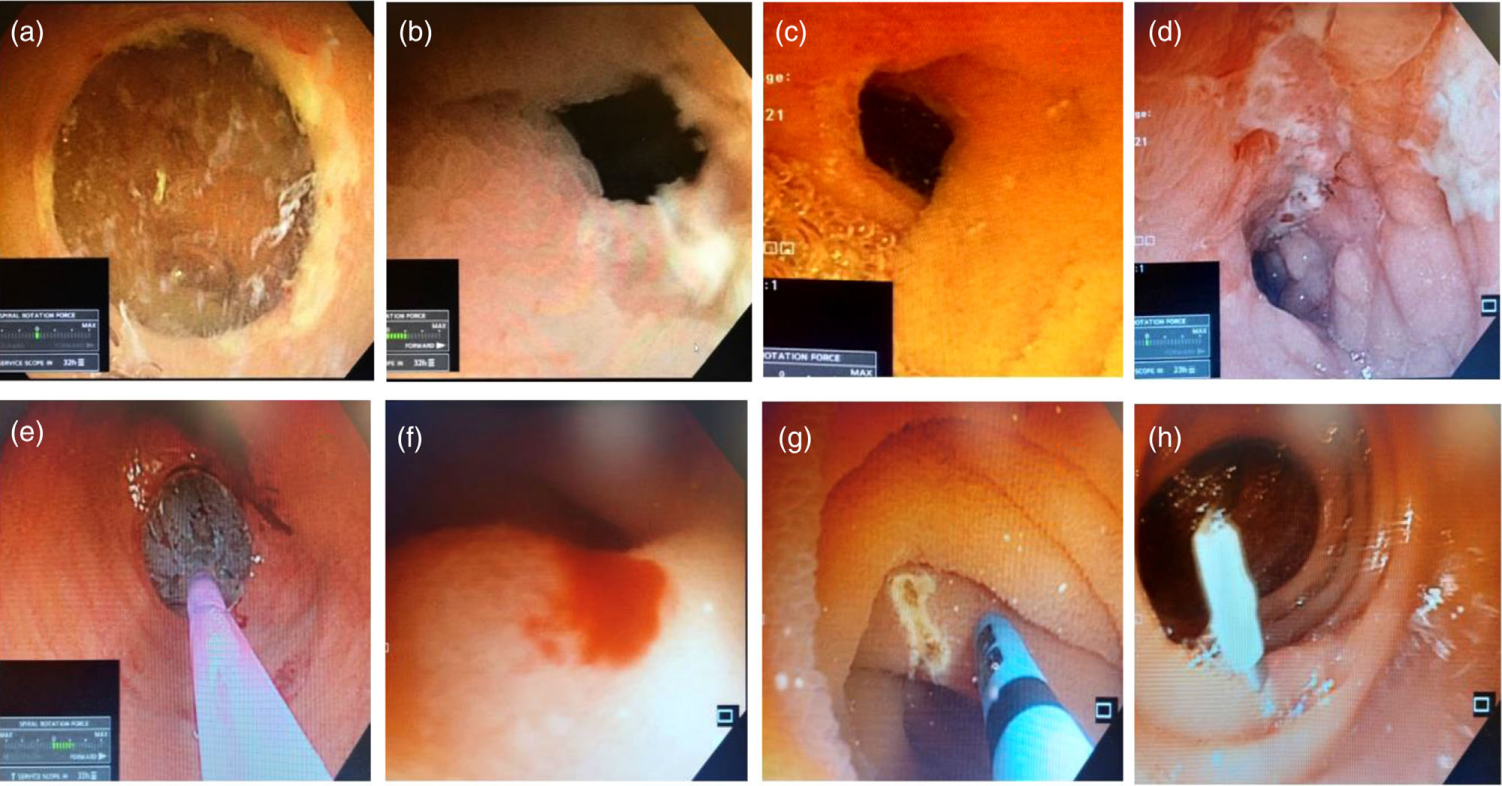
Power spiral enteroscopy images showing (a) mid ileal ulcero‐stricturing lesion, (b) distal ileal ulcer and mucosal edema, (c) proximal ileal stricture, dilated with CRE Balloon, (d) longitudinal, deep serpiginous ulcers with the exudative base forming a cobblestone appearance of mucosa (typically suggestive of Crohn's disease), (e) stricture dilatation using CRE Balloon, (f) jejunal angioectasia with active ooze, (g) bleeding from jejunal angioectasia, treated with argon plasma coagulation, and (h) application of a through the scope clip as a marker for depth of maximal insertion)

**FIGURE 4:**
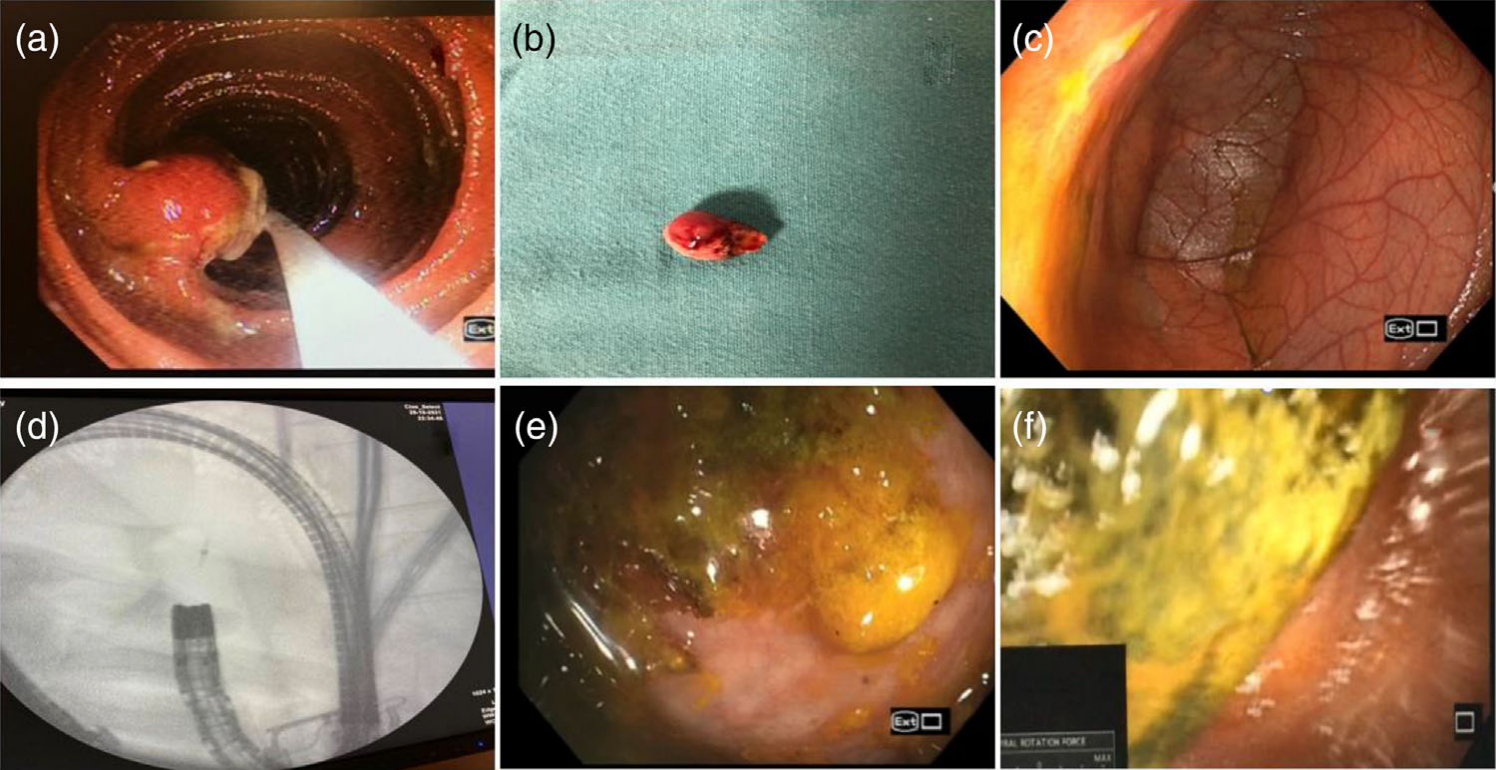
(a) Mid‐jejunal polypoidal lesion seen on antegrade power spiral enteroscopy (PSE), (b) Spiral enteroscope assisted polypectomy specimen of the polypoidal lesion, (c) Meckel's diverticulum with a linear ulcer at its rim, (d) Fluoroscopic image showing spiral enteroscopy in the terminal ileum with clip application just proximal to ileocecal (IC) valve, (e) cecal image on antegrade PSE, and (f) enterolith with jejunal stricture

**FIGURE 5:**
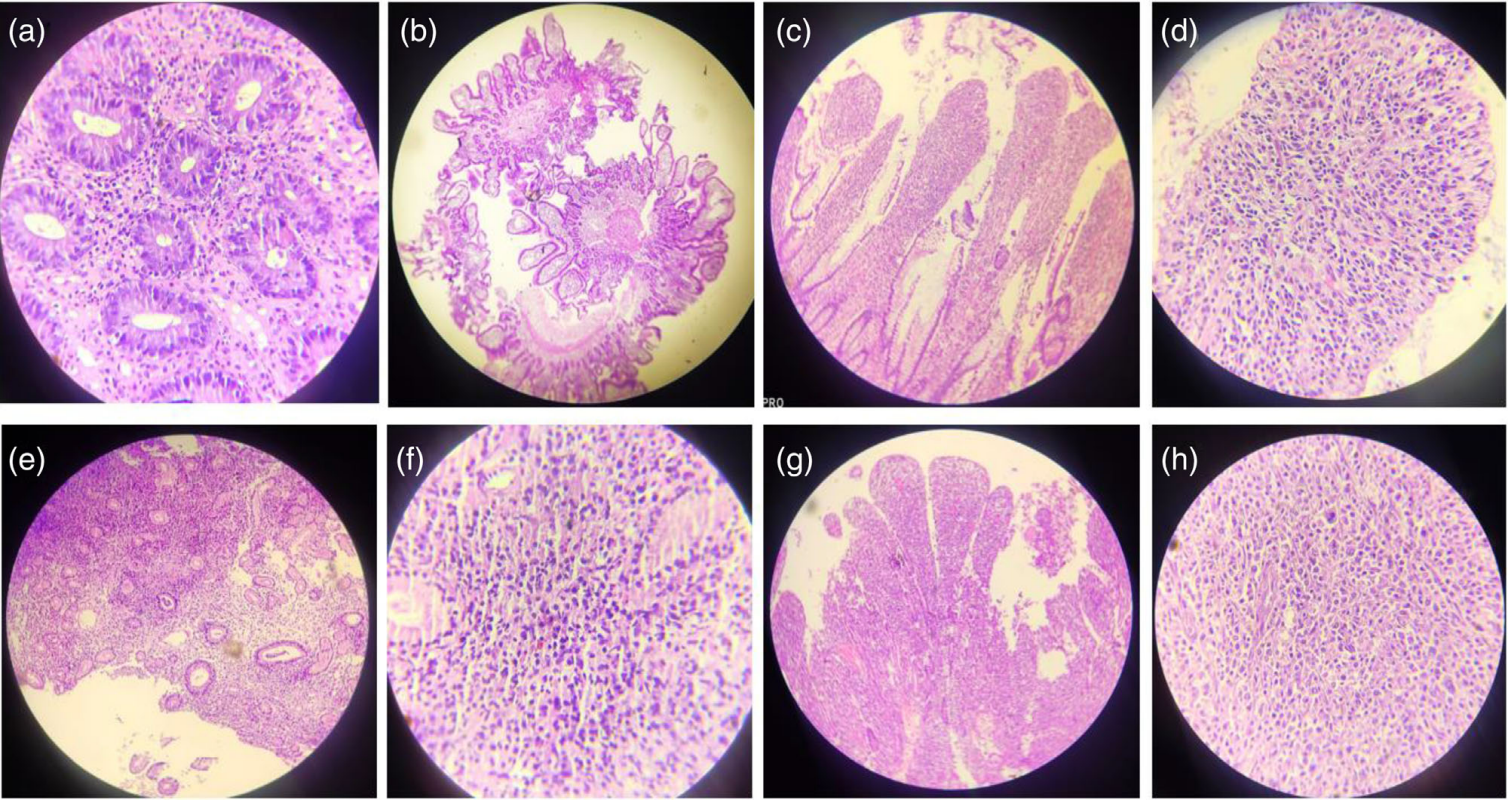
Histopathologic findings: (a) Ileal biopsy showing maintained crypt villous architecture with focal superficial erosions. Lamina propria shows dense lymphoplasmacytic infiltrate suggestive of mild active ileitis (H&E, 40× magnification) (Case 1). (b) Jejunal mucosa with few lymphomononuclear cells in lamina propria, no granuloma or atypia seen suggestive of non‐specific chronic jejunitis (H&E, 4× magnification). (Case 8). (c, d) Histopathological examination of resected specimen (Case 2) shows focal active ileitis with irregularly distributed patchy superficial ulceration and occasional transmural involvement. Features suggestive of Crohn's disease (H&E, 10× and 40× magnification, respectively). (e, f) Jejunal mucosa showing superficial erosions, inflammatory granulation tissue with loss of villi and crypt distortion along with dense lymphoplasmacytic infiltrate in lamina propria. Findings favor Crohn's disease (H&E, 10× and 40× magnification, respectively; Case 9). (g, h) Histopathological examination of resected specimen (Case 6) shows ileal mucosa infiltrated by malignant lymphoid tumor arranged in sheets showing moderate nuclear pleomorphism and vesicular chromatin along with an increase in mitosis and karryohectic debris. These cells are leukocyte common antigen‐positive on initial immunohistochemistry and cytokeratin negative suggestive of lymphoma likely non‐Hodgkin lymphoma (H&E, 10× and 40× magnification, respectively)

Fifty‐four patients (40 males and 14 females) with a mean age of 49.38 ± 16.26 years underwent PSE for various indications (Table 1). A total of 75 PSE procedures in 54 patients were performed (25 antegrade PSE alone, eight retrograde PSE alone, and 21 bi‐directional PSE) (Figure 1). TSR of antegrade and retrograde PSE was 95.55% (43/45) and 93.10% (27/29), respectively. The upper esophageal sphincter could not be negotiated in two patients and the IC valve could not be crossed in two patients. In six patients (four technical failure cases and two patients with esophageal varices) BAE was performed to complete the procedure. The pan‐enteroscopic evaluation was possible in 25 patients (46.29%), and caecum could be reached by antegrade enteroscopy in eight patients. The median total procedure time was 70 min (range 30–110) in the antegrade route and 45 min (range 20–70) in the retrograde approach. Median DMI was 400 cm (range 150–550) in the antegrade route and 180 cm (range 50–350) in the retrograde route. Small bowel ulceration and vascular lesions were the most common findings (Table 2) (Figure 3). Tissue sampling was done in 32/54 patients (59.25%), and Crohn's disease was the most common diagnosis based on histopathology (Figure 5). None of the patients with chronic pain abdomen or chronic diarrhea showed diagnostic findings. The endoscopic therapeutic intervention was performed on 16 patients (therapeutic yield 30.76%). Six patients with active small‐bowel bleed were controlled by APC, and one patient required surgery (Figures 3 and 4). A procedure‐related major adverse event was seen in one patient, who had severe post‐procedural aspiration pneumonitis. Minor adverse events were observed in 20 patients (48.14%). In two patients, duodenal mucosal ooze was seen; both were managed with hemoclip application. Fourteen patients had esophageal mucosal abrasion resulting in odynophagia and sore throat lasting < 72 h, three patients had mild esophageal mucosal tear with hematemesis that resolved spontaneously, and three patients experienced self‐limiting abdominal pain lasting < 48 h (Table 2). Details of each patient undergoing PSE are attached as supplementary text (Table S1)

**TABLE 1:** Baseline patient characteristics and procedure details

**Patients (*n*)**	**54**
Age, year (mean ± SD)	49.38 ± 16.26
Male:female	2.85:1
Indications	
Unexplained chronic abdominal pain with indeterminate findings (doubtful strictures/thickenings) on previous imaging	9 (16.66%)
Recurrent intestinal obstruction with indeterminate findings (normal/doubtful strictures/ thickening) on previous imaging	15 (27.77%)
Suspected small bowel Crohn's disease (vide previous imaging/capsule endoscopy)	6 (11.11%)
Obscure GI bleed/suspected mid‐GI bleed	23 (42.59%)
Chronic diarrhea of unknown cause	1 (1.85%)
ASA	
Grade I	33 (61.11%)
Grade II	17(31.48%)
Grade III	4 (7.4)
Route	
Antegrade	25 (46.29%)
Retrograde	8 (14.8%)
Combined antegrade and retrograde	21 (38.88%)
BAE for failed antegrade/retrograde PSE	6 (11.11%)
Technical success rate	
Antegrade	43/45 (95.55%)
Retrograde	27/29 (93.10%)
Pan‐enteroscopy [TER]	25/54 (46.29%)
Antegrade PSE alone	8 (14.81%)
Antegrade + retrograde PSE/BAE	13 (24.07%)
Retrograde PSE alone	1 (1.8%)
Antegrade BAE + retrograde PSE	3 (5.55%)
Partial enteroscopy	29/54 (53.70%)
Target reached/stricture not negotiable	24 (82.75%)
Target not reached despite maximal possible insertion	3
No specific target and pan‐enteroscopy not attained	2
Total procedure time (min)	
Antegrade, median (range)	70 (30–110)
Retrograde, median (range)	45 (20–70)
Depth of maximal insertion [DMI] (cm)	
Antegrade, median (range)	400 (150–550)
Retrograde, median (range)	180 (50–350)

**TABLE 2:** Diagnostic and therapeutic yields of power spiral enteroscopy in patients with small bowel disorders

**Patients (*n*)**	**54**
Enteroscopic findings (successful cases)	52
Inflammatory lesion (Ulcers ± strictures/strictures)	27 (51.92%)
Vascular lesion (angioectasia/dieulafoy's)	5 (9.61%)
Mass lesion	1 (1.92%)
Polyps	1 (1.92%)
Others: Meckel's diverticulum Ileal Erosions Ascariasis Enterolith Erythema	9 (17.30%) 1 4 1 1 2
Normal	2 (3.84%)
Histopathology	
Crohn's disease	16 (29.62%)
Tuberculosis	1 (1.92)
Non‐specific inflammation	14 (26.92%)
Non‐specific infection	2 (3.84%)
Lymphoma	1 (1.92%)
Lipoma	1 (1.92%)
Normal	1 (1.92%)
Diagnostic yield	46 (85.18%)
Therapeutic interventions	16/52 (30.76%)
APC	6/52 (11.53%)
Stricture dilatation	8 /52 (15.38%)
Injection sclerotherapy	1 (1.92%)
Polypectomy	1 (1.92%)
Adverse events	
Major	1 (1.85%)
Minor	20/54 (48.14%)
Antecedent diagnostic evaluation (total)	158
Endoscopic	
UGI endoscopy/colonoscopy	104
Capsule endoscopy	5
Balloon‐assisted enteroscopy	2
Radiologic	
Small bowel follows through/barium studies	
Abdominal CT (contrast/angiography)	24
Enterography (CT/MRI)	23
Total investigations per subject (Mean ± SD)	3.35 ± 1.23

## DISCUSSION

Limited data is available on the performance and safety of PSE for small bowel evaluation.^9^, ^11^ We demonstrated excellent diagnostic performance of PSE for evaluation of the small bowel with TSR, PER, and diagnostic yield of > 93%, 46.2%, and 85.1% respectively. We could complete the enteroscopic examination with a median total procedure time of 70 min (antegrade route) and 45 min (retrograde route). This is significantly better than the procedure time reported for BAE. In two RCTs comparing double balloon enteroscopy (DBE) and single balloon enteroscopy (SBE), mean procedure times for the combined antegrade and retrograde approach using DBE (161 and 105 min) and using SBE (186 and 96 min), respectively, were considerably longer than the PSE.^10^, ^13^ So, PSE is likely to make the evaluation of small bowel faster.

In our study, technical success was achieved in 95.55% with the antegrade route and 93.10% using the retrograde route. One recent retrospective analysis by Mohan et al.,^11^ using PSE for 61 patients with small bowel disease showed the technical success of 92.85% using the antegrade route and 100% using the retrograde route. Failure with antegrade technique is due to difficult negotiation of the hypopharynx, upper esophageal sphincter, and duodenal‐jejunal flexure, and is likely due to wider enteroscope diameter.

PER is one of the objective parameters that connote the true performance of the procedure. PER in this study was achieved in 46.29%, 14.81% using the antegrade route alone, and 24.07% by bidirectional approach. PER in most recent studies on PSE varies from 10.6% to 60.6% (including our study) and is significantly better than previous enteroscopy techniques (<1% in a recent meta‐analysis of 23 studies with DBE, including 1143 procedures).^12^ Pan‐enteroscopy with BAE is usually achieved with combined bi‐directional enteroscopy (antegrade and retrograde) and is highest for DBE with 40%–80%,^13^, ^14^ substantially lower for SBE (12.4%), and almost nonexistent for spiral enteroscopy (SE, 2.9%).^15^ Achievement of unidirectional pan‐enteroscopy is another biggest advantage with PSE, which could be achieved in eight patients in our study.

In our study, the median DMI was 400 cm (antegrade route) and 180 cm (retrograde route). DMI achieved with PSE compares favorably over those reported with other enteroscopy devices DBE (219 cm, Japanese Multicenter trial), SBE (285 cm), and SE (330 cm).^16^, ^17^ Another important objective parameter of performance is the diagnostic yield of a procedure/tool. A Chinese meta‐analysis by Xin et al.^12^ including 12,823 patients (66 studies/case series) reported an overall diagnostic yield of 68.1% for DBE. It was comparative to the systematic review of 68 trials by Lenz et al.,^15^ who reported a pooled diagnostic yield of 64.4% for DBE, and 53.9% for SBE. The diagnostic and therapeutic yield of 85.18% and 30.76% in our study and other recent studies with PSE Mohan et al. 70% and 24.6% and Beyna et al. 74.2% and 68.2% is clearly better than existing BAE techniques.^9^, ^11^

There is no head‐to‐head comparative study between BAE (DBE and SBE) with PSE or SE with PSE.^18^ BAE is considerably a safer procedure with reported rates of adverse events of about 0.8% for diagnostic procedures and up to 10% for interventions.^19^ In this current study major adverse events are seen in 1.85%; however, the case number is too small to reliably replicate the actual complication rates.

Interestingly, small bowel Crohn's disease was the most common histopathologic finding with 29.62%, In contrast to previous studies, the findings from our study and those reported by Mohan et al.^10^ suggest that small bowel Crohn's disease is not an uncommon etiology of small bowel Ulcero‐stricturing disease in Indian population.^20^ This fact is significant, as it suggests that SB is a predominant small bowel disease, for example, Crohn's disease may remain underdiagnosed due to a lack of appropriate tools.

With the advent of PSE, it is now possible to overcome all the hurdles and limitations of previous BAE in reaching small bowel lesions for acquiring tissue biopsy or achieving hemostasis in mid‐small bowel bleeds. The limitation of our study is a small number of patients, an operator's learning curve, and no comparison with the existing BAE. This is one of the initial studies to assess the efficacy and safety of PSE in patients with small bowel diseases.

To conclude, PSE scores favorably over existing deep enteroscopy devices in terms of procedural ease, duration, safety, and yields and is a promising alternative technique for diagnostic and therapeutic small bowel enteroscopy.

## CONFLICT OF INTEREST

The authors declare no conflict of interest.

## FUNDING INFORMATION

None.

## Supporting information


**Table S1** Details of each patient undergoing Power Spiral Enteroscopy and its findings
