# Supplementary material for: Safety and efficacy of the novel motorized power spiral enteroscopy: A single‐center experience
Source: DEN Open. 2022 Jul 11;3(1):e148. doi: 10.1002/deo2.148 (PMC9307745; doi:10.1002/deo2.148)
Supplement: Supplementary file 1 — Table S1 Details of each patient undergoing Power Spiral Enteroscopy and its findings [file DEO2-3-e148-s001.docx]

Table S1: Details of each patients undergoing Power Spiral Enteroscopy and its findings

| Serial No. | Age (Years) / Sex (M/F) | Indication | Previous Imaging | Route  AG: Antegrade  RG: Retrograde | Successful Procedure (Technical success + Target reached /  Pan-Enteroscopy) | Total procedure time | DMI (Depth of maximal insertion) | Enteroscopic Findings | Histopathologic Findings | Procedure Performed | Adverse Events | Remarks (Diagnostic /Therapeutic yield) |
| --- | --- | --- | --- | --- | --- | --- | --- | --- | --- | --- | --- | --- |
| 1. | 36 / F | Unexplained pain abdomen with indeterminate previous imaging | CTE: Mild thickening in distal ileal loops | AG | Yes | 70 min | 450 cm | AG: Longitudinal deep ulcers in Mid and Terminal ileum | Mild active ileitis not suggestive of Crohn’s | Biopsy | None | Positive: Treated as Non-specific terminal ileitis |
| 2. | 44 /M | Ileal stricture with Anemia / Suspected Crohn’s | CTE: segmental wall thickening, mural hyperenhancement and mural stratification involving distal and terminal ileum | AG + Failed RG-PSE + RG: BAE (Pan-Enteroscopy) | Yes | 90 min+  45 min | 420 cm + 80 cm | AG: Ulcerated stricture at distal ileum. Clip applied  RG-BAE: Stricture reached; single stricture confirmed with visualization of clip across stricture | Highly suggestive of Crohn’s disease | Biopsy | Minor (Esophageal pain) | Positive: Confirmed diagnosis of Crohn’s disease. Underwent surgical management |
| 3. | 24 /M | Suspected Mid GI bleed | CTA: Arterial and delayed enhancing area in distal ileum suggestive of vascular malformation | AG | No | 80 min | 360 cm | AG: Blood clot in distal jejunum (Source of bleed not found) | No | None | None | Negative: Required Surgical intervention |
| 4. | 59 / M | Ileal stricture / Suspected Crohn’s disease | CTE: Jejunal loops are dilated with transition in proximal ileal loops. Mild diffuse symmetric circumferential wall thickening seen at transition zone with preserved mural stratification and partial luminal narrowing. | AG | Yes | 60 min | 220 cm | AG: Proximal ileal stricture with ulceration | Stricturing Crohn’s disease | Biopsy + CRE dilatation (15mm) | Minor (Duodenal ooze) – Clips applied | Positive: Confirmed diagnosis of Crohn’s disease |
| 5. | 57 /F | Chronic diarrhoea with features of malabsorption | CTE: Normal small bowel mucosa | AG: (Pan-enteroscopy: Cecum reached) | Yes | 70 min | 550 cm | AG: Erythema in distal ileum | Unremarkable | Biopsy | Minor (Sore throat, Esophageal pain) | Negative: No specific reason found |
| 6. | 49 / M | Obscure GI bleed |  | AG | Yes | 105 min | 410 cm | AG: Jejunal Angioectasia with active ooze, Ulcerated lesion in proximal ileum | Lymphoma (NHL) | APC done over vascular lesions + Biopsy from ulcerated lesion | Minor (Sore throat and Esophageal pain) | Positive; Confirmed diagnosis of Lymphoma, patient underwent surgery |
| 7 | 34 / M | Unexplained Pain abdomen with Chronic diarrhoea: Inconclusive imaging | CTE: Normal small bowel mucosa | AG | No (No specific target and pan-enteroscopy could not be achieved) | 110 min | 520 cm | AG: Normal mucosa | No | None | None | Negative: No specific reason found |
| 8 | 29 /M | Pain abdomen with ileal thickening on CECT abdomen | CECT: ileal thickening, mesenteric adenopathy | AG + Failed RG PSE, RG BAE (Pan-enteroscopy) | Yes | 90 min | 500 cm | AG: Jejunal Erythema (on PSE), clip applied at distal jejunum.  RG-BAE: Clip reached, distal and terminal Ileal Nodularity | Non-specific jejunitis, Mild Active Ileitis and lymphoid hyperplasia (Non-specific to Crohn’s /TB) | Biopsy | None | Positive: Diagnosis of Non-specific Ileo-jejunitis |
| 9 | 19 /M | Recurrent intestinal obstruction with long segment thickening involving jejunal and ileal loops | MRE: Multifocal circumferential wall thickening involving jejunal and ilea loops | AG | Yes | 60 min | 360cm | AG: Ulcero-stricturing lesion in mid jejunum | Highly suggestive of Crohn’s disease | Biopsy | Minor (Esophageal pain) | Positive: Diagnosis of Crohn’s disease confirmed |
| 10 | 27 / F | Pain abdomen with Ileal stricture on CT scan | CECT: Short segment stricture involving ileum with proximal small bowel distension, surrounding mesenteric haziness and minimal ascites | AG+RG  (Pan-Enteroscopy) | Yes | 110 min +  30 min | 520 cm + 150 cm | AG: Normal till Proximal ileum. Clip applied.  RG distal ileal erosions, clip reached | Non-specific Ileitis | Biopsy | Minor (Duodenal ooze - Clips applied) | Positive: Treated as non-specific terminal ileitis |
| 11 | 60 / M | OGIB | CT: Cirrhosis of liver, no active bleed | AG | Yes | 45 min | 340 cm | AG: Jejunal Angioectasia with active ooze | No | APC done | None | Positive: Hemostasis achieved |
| 12 | 65 /M | Obscure Overt GI bleed | CTE: No significant small bowel abnormality | Antegrade could not be done as scope did not pass beyond neck, Hence BAE done | No | 60 min | 380 cm | Proximal Jejunal small ulcers and Angioectasia with active ooze | No | APC done | Mild superficial esophageal mucosal tear | PSE could not be done |
| 13 | 18 / M | Suspected Crohn’s based on previous imaging | MRE: Multiple skip segments of mural thickening with enhancement causing luminal narrowing involving ileal loops associated with prominent vasa recti | AG + RG | Yes | 110 min + 45 min | 550 cm + 150 cm | AG: Normal till distal ileum, clip applied as mark  RG: distal ileal narrowing with ulceration | Highly suggestive of Crohn’s disease | Biopsy | Minor (Esophageal pain with dysphagia for 2 days) | Positive: Diagnosis of Crohn’s confirmed |
| 14 | 67 / M | Obscure Occult GI bleed with recurrent anemia | CTE: Normal small bowel mucosa | AG | Yes | 60 min | 450 cm | AG; Distal ileal stricture with ulcer | Active ileitis with ulceration | Biopsy + Stricture dilatation | Minor (Pain abdomen ~24 hours with urinary retention) | Positive: In view of lack of other supportive evidences to favour Crohn’s, treated as non-specific Ulcero-stricturing disease |
| 15 | 47 / M | Recurrent partial intestinal obstruction, Jejuno-ileal stricture on CT scan | CTE: Distended jejunal loops, small bowel feces sign and transition zone at jejuno-ileal junction. Terminal jejunal Mural hyperenhancement, engorged vasa recta (Comb sign), perienteric fat stranding and mild ascites. | AG | Yes | 45 min | 320 cm | AG: Distal jejunal stricture | Suggestive of Chronic jejunitis | Biopsy + Stricture dilatation | None | Positive: Treated as Probable Crohn’s based on corroborative imaging findings |
| 16 | 26 / F | Recurrent Partial Intestinal Obstruction, Jejuno-ileal stricture on CT scan | CECT: Dilated proximal small bowel with focal segment bowel wall thickening with mesenteric haze in left flank. Small mesenteric node and mild ascites. | AG+  RG | Yes | 90 min+  50 min | 360 cm + 220 cm | AG: Normal till jejunum, further negotiation not possible, clip applied RG: lleo-jejunal stricture with ulceration | Crohn’s enteritis | Biopsy + Stricture dilatation | None | Positive: Confirmed diagnosis of Crohn’s disease |
| 17 | 24 / F | Recurrent intestinal obstruction with indeterminate imaging | CECT: Small bowel obstruction possibly secondary to adhesions  MRE: Normal small bowel mucosa | AG + RG | Yes | 80 min +  60 min | 460 cm + 180 cm | RG: Normal till mild ileum  AG: proximal ileal stricture | Non-specific Chronic Ileitis | Biopsy + Stricture dilatation | None | Positive: Stricture found and dilated but no definite etiology confirmed |
| 18 | 34 / M | Recurrent intestinal obstruction with indeterminate Imaging (H/o Surgery for appendicular rupture) | MRE: Mildly prominent jejunal loops with no evidence of stratification, thickening, stricture | AG + RG | Yes | 70 min +  20 min | 460 cm + 80 cm | AG: Normal till mid ileum. Retrograde: Terminal ileum stricture |  | Stricture dilatation | None | Positive: terminal ileal stricture (owing to previous surgery) |
| 19 | 37/M | Recurrent Pain abdomen (Suspected Crohn’s) | CTE: Multiple segmental area of ileal thickening with mucosal enhancement and prominent vasa recti. | AG + RG (Pan-enteroscopy) | Yes | 45 min +  40 min | 280 cm + 180 cm | AG: Normal till mid jejunum. Clip applied  RG: Multiple ulcers in mid and distal ileum. Clip reached | Crohn’s Disease | Biopsy | None | Positive: Diagnosis of Crohn’s Confirmed |
| 20 | 42/F | Recurrent unexplained pain abdomen with inconclusive imaging | CECT: Normal | AG (Pan-enteroscopy, Cecum reached) | Yes | 70 min | 540 cm | AG: Normal small bowel mucosa |  | None | Minor: Mild esophageal pain | Negative |
| 21 | 53/M | Recurrent partial intestinal obstruction with jejunal thickening on CT scan | CECT: Mildly dilated jejunal loops with thickening of valvulae and high density stranding of mesenteric fat | AG + RG (Pan-enteroscopy) | Yes | 45 min +  30 min | 250 cm +  200 cm | AG: Mid jejunal ulcers, clip applied  RG: Clip reached (Pan-enteroscopy done) | Non-specific Active jejunitis | Biopsy | None | Positive |
| 22 | 38/M | Recurrent partial intestinal obstruction with indeterminate imaging | CTE: Normal | AG +RG (Pan-enteroscopy) | Yes | 40 min +  45 min | 200 cm +  250 cm | AG: Mid jejunal stricture, clip applied  RG: Normal | Non-specific jejunitis | Biopsy + CRE dilatation | Minor (Esophageal and upper abdominal pain <48 hours) | Positive |
| 23 | 61/M | Obscure overt GI bleed | CTA: Fatty attenuated lesion ~15x11 mm in proximal jejunum | AG | Yes | 80 min | 400 cm | AG: Mid jejunal polypoidal lesion seen and Angioectasia seen in proximal ileum | Submucosal lipoma | Polypectomy + APC of ileal Angioectasia | None | Positive: Averted a surgical intervention |
| 24 | 58/M | Unexplained pain abdomen with indeterminate imaging | MRE: Mucosal hyperenhancement involving terminal ileum and IC junction | AG | Yes | 70 min | 440 cm | AG: Distal and terminal ileal aphthoid ulcers | Active ileitis with ulceration, cryptitis, crypt abscess suggestive possibly of Crohn’s disease | Biopsy | None | Positive: Treated as Crohn’s disease based on corroborative imaging and clinical findings |
| 25 | 54/F | Pian abdomen with chronic diarrhoea suspected small bowel Crohn’s | MRE: Reportedly normal | AG: Pan-enteroscopy | Yes | 90 min | 480 cm | AG: Mid /distal ileal ulcers | Acute on chronic ileitis, transmural inflammation suggestive of Crohn’s disease | Biopsy | Minor (Esophageal pain for 2 days) | Positive: Treated as Crohn’s disease in view of previous imaging and biopsy |
| 26 | 80/M | Recurrent intestinal obstruction with jejunal thickening on CT scan | PET-CT: FDG avid thickening in mid jejunal loops | AG: Failed insertion  AG: BAE: done | NO | 40 min (BAE) | 180 cm | AG BAE: Nodular mucosa in mid jejunum | Giardia infection with Nodular lymphoid hyperplasia | Biopsy | Minor (Esophageal pain) | PSE could not be done |
| 27 | 37/F | Recurrent small bowel obstruction, suspected mid ileal stricture on previous imaging | CTE: Short segment circumferential bowel wall thickening in mid ileum with partial obliteration of wall layer stratification | AG +RG | Yes | 60 min +  40 min | 360 cm +  100 cm | AG: Seen up to proximal ileum: Normal mucosa, Clip applied  RG: Focal mid ileal stricture with ulceration | Distorted crypt architecture, cryptitis, mixed inflammatory cell infiltrate suggestive of Crohn’s disease | Biopsy | Major (Aspiration pneumonitis, requiring ICU admission) | Positive: Treated as Crohn’s disease based on corroborative imaging findings |
| 28 | 45/M | Obscure overt GI bleed | CTA: Normal | AG +RG (Pan-enteroscopy) | Yes | 50 cm +  30 min | 300 cm +  180 cm | AG: Normal up to proximal ileum, clip applied  RG: Clip reached, Meckel’s diverticula with linear ulceration at its rim noted |  | None | None | Positive: Cause of bleed found, referred for surgical intervention |
| 29 | 78/M | Obscure overt GI bleed | CTA- Normal | AG: Pan-enteroscopy | Yes | 90 min | 520 cm | AG: Cecum reached; distal ileal erosion seen (NSAIDS related) |  | None | None | Positive: Cause of bleed found |
| 30 | 30/M | Pain abdomen with diarrhoea, Suspected Crohn’s disease | CTE: Normal | AG: Pan-enteroscopy | Yes | 80 min | 500 cm | AG: Cecum reached, Distal ileal ulcer | Benign lymphoid hyperplasia, no cryptitis, crypt abscess | Biopsy | Minor (Esophageal pain <48 hours) | Positive: Not treated as Crohn’s, treated as nonspecific inflammation |
| 31 | 54/M | Unexplained pain abdomen, Suspected Crohn’s disease | MRE: Normal  Capsule Endoscopy: Multiple ileal ulcers | AG: Pan-enteroscopy | Yes | 90 min | 550 cm | AG: Multiple mid/distal ileal ulcers seen, reached up to Terminal ileum just proximal to IC valve (Confirmed on fluoroscopy) | Ulceration with inflammatory granulation tissue suggestive of non-specific infection | Biopsy | None | Positive: Treated as non-specific infection /inflammation |
| 32 | 40/M | Unexplained pain abdomen with indeterminate findings on previous imaging | CTE: Normal | AG+ RG (Pan-enteroscopy) | Yes | 50 min+  40 min | 280 cm +  250 min | AG: Normal up to mid jejunum, clip applied  RG: Clip reached, Panenteroscopy achieved. Ascariasis seen in mid small bowel |  | None | None | Positive: Cause of pain found: Worm infestation |
| 33 | 66/M | Recurrent Small bowel obstruction | CTE: distended Jejunum with multiple segments of luminal narrowing in mid/distal small bowel associated with mesenteric Lymphadenopathy, mild ascites | RG | Yes | 45 min | 150 cm | RG: Mid ileal stricture with ulceration | Non-specific ileitis | Biopsy | None | Positive: Treated as Tubercular cause based on past h/o Pulmonary TB and imaging findings |
| 34 | 65/M | Obscure GI bleed | CTE: Mid ileal stricture | AG +RG | No | 60 min +  20 min | 220 cm +  50 cm | AG: seen up to mid jejunum, small bowel lymphangiectasia  RG: seen up to 50 cm, normal mucosa |  | None | None | Negative: Mid ileal stricture seen on CTE could not be reached |
| 35 | 68/M | Obscure overt GI bleed | NCCT abdomen: Mild wall thickening in distal ileal region up to ileocolic region | AG + RG (Pan-enteroscopy) | Yes | 45 min +  40 min | 150 cm +  200 cm | AG: Jejunal stricture with ulceration, Enterolith proximal to stricture  RG: Normal up to distal jejunum | Non-specific jejunitis | Biopsy | Minor (Esophageal pain and bleed for 2 days) | Positive: Stricture reached and cause of bleed found |
| 36 | 58/ M | Obscure overt GI bleed | CTA: Portal hypertension, peri-splenic collaterals  Capsule Endoscopy: Small bowel ulcers | RG in view of Esophageal varices | Yes | 60 min | 250 cm | RG: Multiple telangiectasia in SI till distal jejunum |  | APC done | No | Positive: Cause of bleed found; Endo therapy done |
| 37 | 69 /M | Obscure overt GI bleed | CTA: Area of jejunal ooze  Capsule Endoscopy: Small bowel bleed | RG in view of Esophageal varices  AG: evaluation by BAE  Pan-enteroscopy achieved | Yes | 50 min | 220 cm | RG: Previously placed clip by antegrade BAE reached |  | None | None | Positive: Pan-enteroscopy done |
| 38 | 50/M | Obscure Overt GI bleed | CTA: Abnormal wall thickening/ enhancement in terminal ileum, fat stranding, ascites | RG | Yes | 60 min | 150 cm | RG: Distal ileal ulcer | Focal acute ileitis | Biopsy + Injection Sclerotherapy done | None | Positive: Source of bleeding found |
| 39 | 32/F | Recurrent Partial intestinal obstruction with doubtful stricture | CTE: Jejunal thickening | AG | Yes | 45 min | 150 cm | AG: Proximal jejunal stricture with ulceration | Focal cryptitis, inflammatory granulation tissue, dense mixed inflammatory cells suggestive of active jejunitis | Biopsy | Minor (Esophageal pain mild lasting 1 day) | Positive: Treated as Crohn’s disease |
| 40 | 64/M | Recurrent obstruction with previous imaging suggestive of Ileal stricture | CTE: Four areas of mucosal thickening with hyperenhancement in proximal, mid, distal ileum with mild narrowing | AG: Pan-enteroscopy | Yes | 60 min | 450 cm | AG: Jejunal and Proximal ileal ulcers | Lymphoid follicular hyperplasia with cryptitis, crypt distortion and abscess | Biopsy | Minor (Esophageal pain for 2 days) | Positive: Treated as Crohn’s disease |
| 41 | 52/F | Obscure overt GI bleed | CTE: Normal | AG + RG | Yes | 65 min +  40 min | 200 cm + 150 cm | AG: Jejunal erythema  RG: Ileal erosions |  | None | Minor (Mucosal abrasions in esophagus) | Positive: Possible cause for bleed was ileal erosions |
| 42 | 63/M | Recurrent intestinal obstruction with indeterminate previous imaging | CECT abdomen: Normal | AG: Pan-enteroscopy | Yes | 70 min | 430 cm | AG: Distal jejunal and ileal aphthoid ulcers | Focal cryptitis, crypt abscess with preserved crypt architecture suggestive of focal active enteritis | Biopsy | Minor (Esophageal pain and discomfort for 2 days) | Positive: Non-specific acute enteritis |
| 43 | 51/M | Obscure overt GI bleed | CTA: ? GIST mid ileal | RG + AG (Pan-enteroscopy) | Yes | 30 min  45 min | 300 cm +  150 cm | RG: Seen up to proximal ileum, normal mucosa, clip applied  AG: Clip reached, pan-enteroscopy completed |  | None | None | Positive: Extraluminal lesion confirmed after pan-enteroscopy |
| 44 | 60/F | Recurrent small bowel obstruction with imaging suggestive of ileal and jejunal strictures | MRE: multifocal short segment strictures in small bowel loops with intermittent bowel dilatation | AG + RG | Yes | 40 min +  30 min | 200 cm +  150 cm | AG: Stricture with circumferential ulcer seen in mid jejunum, clip applied  RG: Seen up to mid ileum, small discrete ulcers | Lamina propria infiltrate seen with maintained crypt architecture suggestive of focal active jejunitis | Biopsy + CRE dilatation (18 mm) | None | Positive: Treated as non-specific jejunitis |
| 45 | 23/F | Obscure occult GI bleed | CTA: Normal | RG + AG (Pan-enteroscopy) | Yes | 30 min +  60 min | 150 cm +  400 cm | RG: Normal till mid ileum, clip applied  AG: Clip reached; mucosa normal |  | None | None | Positive: Pan-enteroscopy done, no definite cause for bleed found |
| 46 | 74/M | Obscure GI bleed | CTA: Normal | RG +AG (Pan-enteroscopy) | Yes | 60 min +  40 min | 300 cm + 150 cm | RG: Seen up to proximal/ mid jejunum, clip applied, normal mucosa  AG: Clip reached |  | None | None | Positive: Pan-enteroscopy done |
| 47 | 38/F | Obscure GI bleed | CECT: Small bowel thickening | RG + AG (Pan-enteroscopy) | Yes | 50 min +  45 min | 250 cm + 200 cm | RG: Normal up to mid jejunum, clip applied  AG: Clip reached |  | None | None | Positive: Pan-enteroscopy done |
| 48 | 56/F | Pain abdomen with indeterminate previous imaging | CTE: Mild wall thickening in mid small bowel | RG: Pan-enteroscopy | Yes | 70 min | 450 cm | RG: Seen, up to DJ flexure, white based ulcers seen in distal jejunal and proximal ileum | Crohn’s Enteritis | Biopsy | None | Positive: Diagnosed as Crohn’s disease |
| 49 | 56/M | Partial intestinal obstruction, previous imaging suggestive of ileal stricture | CTE: Segments of abnormal wall enhancement and thickening with luminal narrowing in terminal ileum | AG | Yes | 80 min | 400 cm | AG: Normal up to distal ileum |  | None | None | Negative: Strictures described on CT can’t be reached |
| 50 | 79/M | Obscure GI bleed | CTA: Normal | AG: Pan-enteroscopy completed ileo-colonoscopy | Yes | 75 min | 430 cm | AG: Normal mucosa up to distal ileum, clip applied. Clip reached with ileo-colonoscopy |  | None | Minor (Mild esophageal pain) | Positive: Pan-enteroscopy done |
| 51 | 38/M | Obscure overt GI bleed, underlying CLD | CTA: Cirrhosis of liver, portal hypertension, normal small bowel | RG: In view of esophageal varices  AG: Evaluation by BAE | Yes | 50 min | 320 cm | RG: Normal up to proximal jejunum, clip applied  AG: by BAE clip reached |  | None | None | Positive: Pan-enteroscopy done |
| 52 | 59/M | Obscure GI bleed | CTA: Segment of mural thickening involving ileal loops in left lower abdomen with prominent vasa recti and prominent mural vessels | RG | Yes | 60 min | 150 cm | RG: Seen up to distal jejunum, circumferential ileal ulcer | Focal active ileitis, crypt distortion, cryptitis and crypt abscess suggestive of Crohn’s disease | Biopsy | None | Positive: Diagnosed as Crohn’s disease |
| 53 | 52/M | Obscure GI bleed | CTE: Multiple skip areas of wall thickening and slight mural stratification involving mid/distal small bowel loops | AG | Yes | 70 min | 410 cm | AG: Seen up to distal ileum, stricture with circumferential ulceration seen, second non-negotiable stricture seen 5cm beyond first | Suggestive of Crohn’s disease | Biopsy | Minor (Mild esophageal pain) | Positive: Diagnosed as Crohn’s disease |
| 54 | 65/F | Obscure GI bleed, underlying CLD | Capsule Endoscopy: Multiple small bowel angio-ectasia  CTA: Normal small bowel | RG: In view of esophageal varices  AG: Evaluation by BAE | Yes | 50 min | 350 cm | RG: Seen up to mid jejunum, ileal angio-ectasia seen. Clip applied  AG BAE: Clip reached, Pan- enteroscopy done |  | APC done over ileal angio-ectasia | None | Positive: Cause of bleed found and endotherapy done |
